# Refinement for single-nanoparticle structure determination from low-quality single-shot coherent diffraction data

**DOI:** 10.1107/S2052252519014222

**Published:** 2020-01-01

**Authors:** Toshiyuki Nishiyama, Akinobu Niozu, Christoph Bostedt, Ken R. Ferguson, Yuhiro Sato, Christopher Hutchison, Kiyonobu Nagaya, Hironobu Fukuzawa, Koji Motomura, Shin-ichi Wada, Tsukasa Sakai, Kenji Matsunami, Kazuhiro Matsuda, Tetsuya Tachibana, Yuta Ito, Weiqing Xu, Subhendu Mondal, Takayuki Umemoto, Christophe Nicolas, Catalin Miron, Takashi Kameshima, Yasumasa Joti, Kensuke Tono, Takaki Hatsui, Makina Yabashi, Kiyoshi Ueda

**Affiliations:** a Division of Physics and Astronomy, Kyoto University, Kyoto 606-8501, Japan; b RIKEN SPring-8 Center, Sayo, Hyogo 679-5148, Japan; c Linac Coherent Light Source, SLAC National Accelerator Laboratory, Menlo Park, CA 94025, USA; d Chemical Sciences and Engineering Division, Argonne National Laboratory, 9700 S. Cass Avenue, Lemont IL 60439, USA; e Paul-Scherrer Institute, CH-5232 Villigen PSI, Switzerland; f LUXS Laboratory for Ultrafast X-ray Sciences, Institute of Chemical Sciences and Engineering, École Polytechnique Fédérale de Lausanne (EPFL), CH-1015 Lausanne, Switzerland; g Institute of Multidisciplinary Research for Advanced Materials, Tohoku University, Sendai 980-8577, Japan; h Department of Physical Science, Hiroshima University, Higashi-Hiroshima 739-8526, Japan; i Synchrotron SOLEIL, l’Orme des Merisiers, Saint-Aubin, BP 48, 91192 Gif-sur-Yvette Cedex, France; j Extreme Light Infrastructure - Nuclear Physics (ELI-NP), "Horia Hulubei" National Institute for Physics and Nuclear Engineering, 30 Reactorului Street, Măgurele RO-077125, Jud.Ilfov, Romania; k LIDYL, CEA, CNRS, Université Paris-Saclay, CEA Saclay, 91191 Gif-sur-Yvette, France; l Japan Synchrotron Radiation Research Institute (JASRI), Sayo, Hyogo 679-5198, Japan

**Keywords:** coherent diffractive imaging, phase problem, single particles, XFELs, structure reconstruction, computation, clusters, electron density

## Abstract

With the emergence of X-ray free-electron lasers, it is possible to investigate the structure of nanoscale samples by employing coherent diffractive imaging in the X-ray spectral regime. In this work, we developed a refinement method for structure reconstruction applicable to low-quality coherent diffraction data. The method is based on the gradient search method and considers the missing region of a diffraction pattern and the small number of detected photons. We introduced an initial estimate of the structure in the method to improve the convergence. The present method is applied to an experimental diffraction pattern of an Xe cluster obtained in an X-ray scattering experiment at the SPring-8 Angstrom Compact free-electron LAser (SACLA) facility. It is found that the electron density is successfully reconstructed from the diffraction pattern with a large missing region, with a good initial estimate of the structure. The diffraction pattern calculated from the reconstructed electron density reproduced the observed diffraction pattern well, including the characteristic intensity modulation in each ring. Our refinement method enables structure reconstruction from diffraction patterns under difficulties such as missing areas and low diffraction intensity, and it is potentially applicable to the structure determination of samples that have low scattering power.

## Abstract 2

A refinement method of the structure from a low-intensity diffraction pattern is proposed and applied to a diffraction pattern from a sub-micrometre cluster. It is shown that the method could retrieve a 2D projection of the electron density that is physically meaningful.

## 1. Introduction   

The recent advent of X-ray free-electron lasers (XFELs) has offered new opportunities to investigate the structure of a single nanoscale sample by using a shot-by-shot X-ray diffraction method on the basis of the diffraction-before-destruction scheme (Neutze *et al.*, 2000 ▸). Pioneering works have demonstrated the advantage of XFELs for determining the structure of biomolecules (Chapman *et al.*, 2011 ▸; Seibert *et al.*, 2011 ▸; Kimura *et al.*, 2014 ▸; van der Schot *et al.*, 2015 ▸) and non-biological and fragile objects (Bogan *et al.*, 2008 ▸; Bostedt *et al.*, 2010 ▸; Xu *et al.*, 2014 ▸; Gomez *et al.*, 2014 ▸).

For fundamental questions on ultrafast X-ray scattering, atomic clusters have been widely used as model systems because of their simple electronic and geometric structure. Their geometric structure can be well characterized with X-ray imaging (Bostedt *et al.*, 2010 ▸; Rupp *et al.*, 2012 ▸, 2014 ▸; Gomez *et al.*, 2014 ▸; Barke *et al.*, 2015 ▸), and the interaction of intense light pulses with clusters has been characterized from the optical (Ditmire *et al.*, 1996 ▸, 1997 ▸) to the vacuum ultraviolet (Wabnitz *et al.*, 2002 ▸; Bostedt *et al.*, 2008 ▸) and X-ray spectral regime (Bostedt *et al.*, 2012 ▸; Gorkhover *et al.*, 2012 ▸; Ferguson *et al.*, 2016 ▸). The first applications of coincident X-ray imaging and spectroscopy showed great advantages in obtaining geometric and electronic structure at the same time. With this approach it was possible to eliminate the effects of the cluster size distribution as well as the focal volume intensity distribution on the physical processes, and a size-structural-selective evaluation of the processes was realized (Gorkhover *et al.*, 2012 ▸, 2016 ▸).

Coherent diffractive imaging (CDI) in the X-ray spectral regime (Miao *et al.*, 1999 ▸, 2015 ▸ Chapman *et al.*, 2006 ▸) is a promising method to explore the structure of single particles by recording diffraction patterns with single exposures to XFEL pulses. CDI methods enable us to obtain the information for an electron-density map of the samples with high spatial resolution because of short-wavelength X-ray pulses.

In CDI, reconstruction of a structure from diffraction patterns is achieved with suitable phase retrieval (PR) algorithms. In PR algorithms, the phase is recovered iteratively by applying constraints in the Fourier space and the real space. The error reduction (ER) algorithm (Fienup, 1982 ▸) is one of the well known algorithms but suffers from stagnation issue at local minima. More advanced algorithms such as the hybrid input-output (HIO) algorithm (Fienup, 1982 ▸) and the relaxed averaged alternating reflections (RAAR) algorithm (Luke, 2005 ▸) have been proposed. These advanced algorithms converge faster than the ER algorithm in many cases and also present the advantage that they can efficiently escape from local optima. Several studies (Takahashi *et al.*, 2013 ▸; Kimura *et al.*, 2014 ▸; van der Schot *et al.*, 2015 ▸; Tanyag *et al.*, 2015 ▸) have employed iterative PR algorithms to coherent diffraction patterns and demonstrated the feasibility of structure reconstruction of single particles. Much effort has been devoted to obtaining high-quality diffraction patterns.

However, in practice it is often challenging to obtain high-quality diffraction patterns that can be successfully phased with the existing algorithms as there are some problems applying the PR algorithms to experimental diffraction patterns. One of the problems is the missing area of the detector in the small-angle region (Martin *et al.*, 2012*a* ▸; Kobayashi *et al.*, 2014 ▸). In CDI measurements, the central part of the diffraction patterns is usually not recorded because of a direct beamstop which protects the detector. The small-angle region contains information about the overall shape of the object and previous studies (Li *et al.*, 2017 ▸) show that the loss of the central part is sometimes critical for PR. It is also reported by Thibault *et al.* (Thibault *et al.*, 2006 ▸) that missing data cause very weakly constrained degrees of freedom, called unconstrained modes, which are not restricted by the available information in the real and Fourier space. These modes give rise to the ambiguity of reconstruction and some studies show that removal of this ambiguity leads to a better convergence to structural reconstruction using PR (Thibault *et al.*, 2006 ▸; Seibert *et al.*, 2011 ▸).

Another problem is the limited number of diffracted photons from single particles. This is especially true for biomolecules as the diffracted intensity from biomolecules is usually much smaller than that from heavier atom metal or semiconductor nanoparticles. Diffraction patterns with a small number of photons contain noise because of photon-counting statistics, which limits the resolution of the reconstructed structure (Martin *et al.*, 2012*b* ▸). As an alternative analysis to iterative PR, one can directly discuss the diffraction patterns quantitatively (Li *et al.*, 2017 ▸) or construct structural models that reproduce the diffraction patterns (Langbehn *et al.*, 2018 ▸). However, these analyses cannot eliminate the arbitrariness in structure modeling, and significant structural information in the diffraction patterns might be overlooked.

In this article, we describe a refinement method to reconstruct the structure from low-quality diffraction patterns. The structure is refined from an initial estimate by using a likelihood function that considers both the missing region of the detector and the statistical errors of the intensity. We apply the method to a diffraction pattern from a giant Xe cluster recorded at the SPring-8 Angstrom Compact Free-Electron Laser (SACLA) facility (Ishikawa *et al.*, 2012 ▸).

## 2. Experiment   

The experiment was carried out at experimental hutch 3 of beamline 3 at SACLA (Tono *et al.*, 2013 ▸). The schematic of the experimental setup has been described elsewhere (Fukuzawa *et al.*, 2018 ▸). The wavelength of the XFEL was set to 2.2 Å (corresponding to a photon energy of 5.5 keV). The XFEL pulses were focused with a Kirkpatrick–Baez (KB) mirror system to a focus size of 1.5 × 1.3 µm (full width at half-maximum), resulting in a typical peak fluence of ∼16 µJ µm^−2^. The stray light from the edge of the KB mirrors was reduced using an aperture made of a 0.5 mm thick tungsten plate, equipped with a 0.3 × 0.3 mm square aperture and installed 200 mm upstream of the interaction region. Xe clusters were prepared by expanding Xe gas adiabatically through a pulsed valve attached to a convergent–divergent nozzle with a diameter of 200 µm and a half angle of 4°. The stagnation pressure and temperature were 21 bar and 300 K, respectively. In the experiments, the timing of valve opening was adjusted so that the after-pulse region of the gas jet where extremely large clusters were generated, which had been observed in a preceding study (Rupp *et al.*, 2014 ▸), arrived at the interaction point at the X-ray pulse arrival time.

The shot-by-shot scattering signals were recorded with a multiport charge-coupled device (MPCCD) octal sensor (Kameshima *et al.*, 2014 ▸). The detector covered a scattering angle range of 0.10–2.8°, which corresponds to a momentum transfer of *q* = 0.049–1.4 nm^−1^.

**Figure 1:**
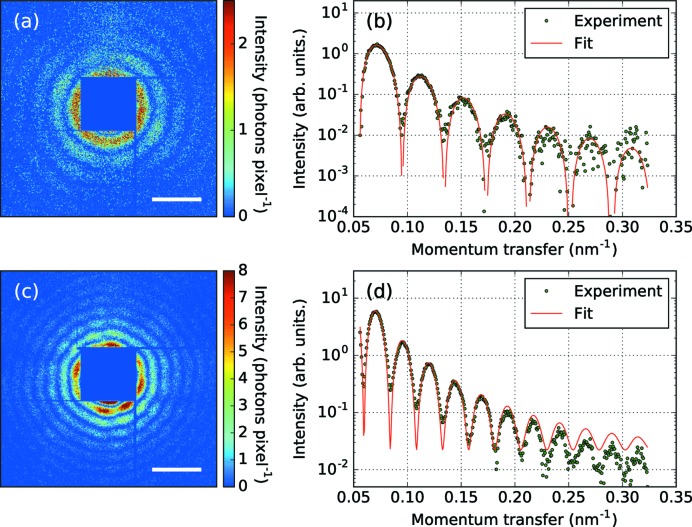
Measured diffraction patterns from Xe clusters. (*a*) A concentric ring pattern suggesting a spherical cluster. (*b*) The 1D scattering intensity deduced from the pattern (*a*) and a fitting curve. (*c*) A pattern consisting of concentric rings with aperiodic intensity modulation suggests existence of a cluster having surface structures. (*d*) The 1D scattering intensity deduced from the pattern (*c*) and a fitting curve. The scale bars in (*a*) and (*c*) are 0.1 nm^−1^.

Figs. 1 ▸(*a*)–1 ▸(*d*) show diffraction patterns of Xe clusters recorded with the octal MPCCD sensor. The central region of the diffraction patterns is missing because of the intense stray light signal from upstream X-ray optics. Most of the obtained diffraction patterns consist of concentric rings corresponding to spherical clusters [Fig. 1 ▸(*a*)]. In addition, there were a few patterns suggesting spheroidal clusters and interference patterns associated to two particles being present in the focus, as reported in earlier works (Bostedt *et al.*, 2010 ▸; Rupp *et al.*, 2012 ▸; Gorkhover *et al.*, 2018 ▸).

In the following sections, we focus on the brightest pattern obtained in the present experiment [Fig. 1 ▸(*c*)], which is still scarce in photon count compared with most other studies at longer wavelength or with larger structures. The diffraction pattern shows characteristic concentric rings with aperiodic intensity modulation in the circumferential direction of each ring, which indicates that the cluster has some structure on its surface (Bostedt *et al.*, 2012 ▸; Rupp *et al.*, 2014 ▸). The 1D scattering intensity deduced from circumferential integration of this image was fitted using the scattering intensity of a uniform dense sphere, as shown in Fig. 1 ▸(*d*), giving a cluster radius of 129 nm.

As mentioned above, there are several difficulties for reconstructing the structure from a diffraction pattern with iterative PR algorithms. These are especially prominent for this pattern of a small structure in hard X-ray pulses. One of the problems is the large missing region around the center of the detector. In fact, the diffraction pattern loses as much as 99% of the diffracted photons. The total number of detected photons was only ∼73 000. Another issue is the statistical error of the intensity caused by photon counting. The number of photons was <10 per pixel in the central side of the image and <1 per pixel at *q* = 0.3 nm^−1^. The Shannon pixel, defined by Ω_s_ = λ^2^/(4*w* ^2^) (Huldt *et al.*, 2003 ▸; Ho *et al.*, 2016 ▸) where λ is the wavelength of the incident photons and *w* = 2*r*_0_ is the diameter of the observed cluster (*r*_0_ is the cluster radius), included ∼170 detector pixels, and the number of photons per Shannon pixel at *q* = 0.3 nm^−1^ was ∼3 . We attempted to apply ER and HIO algorithms to the diffraction pattern shown in Fig. 1 ▸(*c*) but no physically reasonable structure could be obtained using these algorithms. In order to overcome the limitations of the established approaches for weakly scattering samples we developed the following refinement method.

## 3. Analysis   

### 3.1. Refinement method   

In this study, we used the gradient search method (Fienup & Wackerman, 1986 ▸) and accounted for the missing region of the detector and the small number of photons, as described in previous work (Ikeda & Kono, 2012 ▸), and combined this method with an initial estimate of the structure.

We neglected absorption in light of the thin sample and high photon energies and considered a positive-valued 2D projected electron density 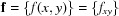. The discrete Fourier transform of the electron density **f** is defined as follows,
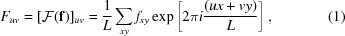
where (*u*, *v*) is the 2D index in the Fourier space and *L* × *L* is the dimension of the matrix. The intensity 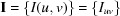 is proportional to the square of the Fourier modulus,
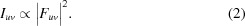
We denote the number of detected photons as 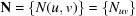. Basically, PR is an optimization problem in which an electron density that best reproduces the experimental data is searched, subject to a certain constraint. Widely used algorithms such as ER and HIO algorithms maximize the Gaussian likelihood (*i.e.* minimize the residual sum of squares),
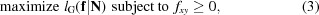


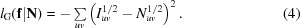
However, the intensity recorded in CDI measurements largely suffers from Poisson noise because of photon-counting statistics. Hence, we decided to use the Poisson likelihood 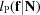 as the objective function for the optimization, which has also been used to refine the density map obtained from a PR algorithm (Thibault & Guizar-Sicairos, 2012 ▸),
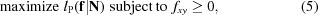


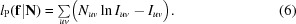
We carried out optimization of the Poisson likelihood with a conventional gradient descent method. The gradient of the Poisson likelihood function is expressed as follows (Ikeda & Kono, 2012 ▸),
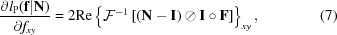
where 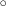 and 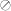 represent the element-wise multiplication and division of matrices, respectively. Using the gradient, the electron density is updated in each step of iteration as follows,
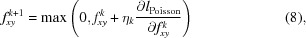
where η_*k*_ is the step size. The determination of the step size is important for rapid convergence of the optimization as well as for successful reconstruction of structures. In computation, we used the line-search function line_search built in the *SciPy* module for Python to search for the step size satisfying the strong Wolfe conditions (Wolfe, 1969 ▸) with its parameters *c*_1_ = 0.0001 and *c*_2_ = 0.9 by following Nocedal and Wright (Nocedal & Wright, 1999 ▸).

For diffraction data with missing regions, the Poisson likelihood function is modified as follows,
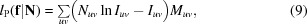
where 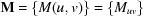 is a mask that specifies the missing regions of the detector with a value of 1 where data are obtained and a value of 0 where data are missing. In the present analysis, we used a mask corresponding to the missing region around the center and the gaps in the assembled detector. The gradient of the modified Poisson likelihood is expressed as follows,
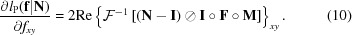
In general, when one solves a non-convex optimization problem with a gradient search method, the choice of the initial structure crucially affects the result of the optimization. Inappropriate choice of the initial structure leads to stagnation of the optimization at local optima. The present method cannot resolve this stagnation problem, which potentially leads to model-biased results strongly dependent on the initial estimate/model. Therefore, we need to construct a good initial estimate of the structure before starting the iteration, and from this point the present method can only be used for the refinement of the initial estimate.

### 3.2. Structure reconstruction from simulation data   

**Figure 2:**
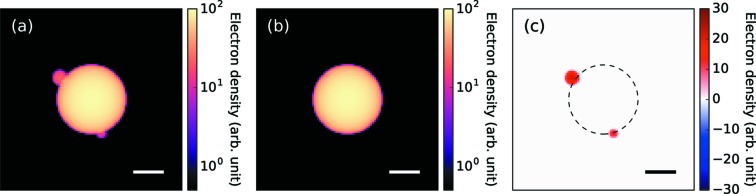
The object used in the simulation of the structure reconstruction. (*a*) 2D projected electron density of the object. (*b*) 2D projection of a uniform sphere used as the initial structure in structure optimization. (*c*) Difference between (*a*) and (*b*) defined as (*a*) − (*b*). The scale bars are 100 nm.

**Figure 3:**
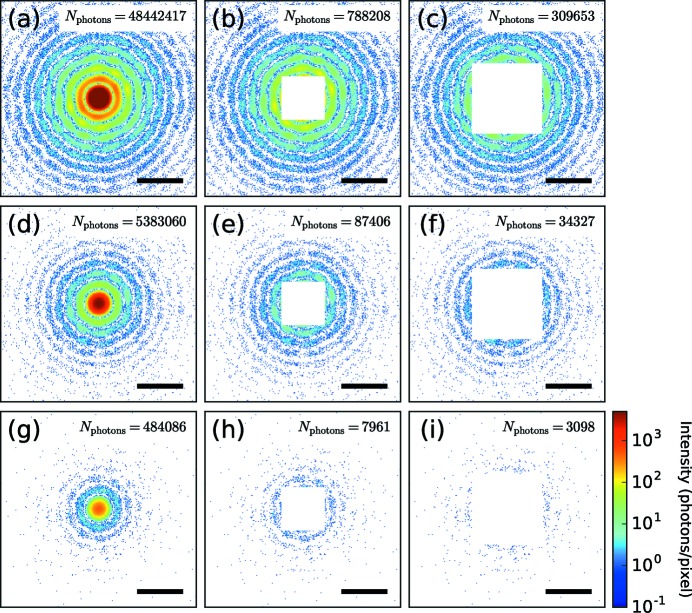
Diffraction patterns simulated from the electron density shown in Fig. 2 ▸(*a*). The top row represents the highest incident XFEL fluence case and the fluence is reduced as it moves to the bottom. The first column shows the diffraction patterns without missing regions and the missing region becomes larger as it moves to the right. The current experimental condition corresponds to the middle one (*e*). The scale bars are 0.1 nm^−1^.

To evaluate the effectiveness of the structure refinement method, we first applied the method to simulated data. In the simulation, we prepared diffraction patterns from a spherical cluster with two small spheres attached to its surface [Fig. 2 ▸(*a*)], similar to previous experimental studies (Rupp *et al.*, 2012 ▸). Three different incident XFEL fluences and three different levels of the missing region of the detector were respectively assumed. The calculated diffraction patterns are shown in Fig. 3 ▸. We started the structure refinement with a uniform dense sphere [Fig. 2 ▸(*b*)] and the electron density was updated iteratively according to the equations (8) and (10).

**Figure 4:**
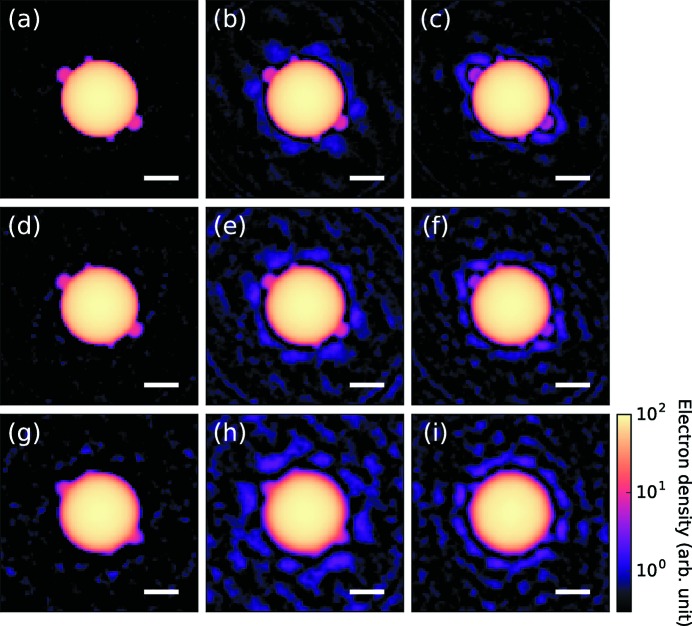
Electron density reconstructed from the simulation data using the present method. Each figure represents the reconstructed structure obtained from the corresponding diffraction pattern in Fig. 3 ▸. The scale bars are 100 nm.

**Figure 5:**
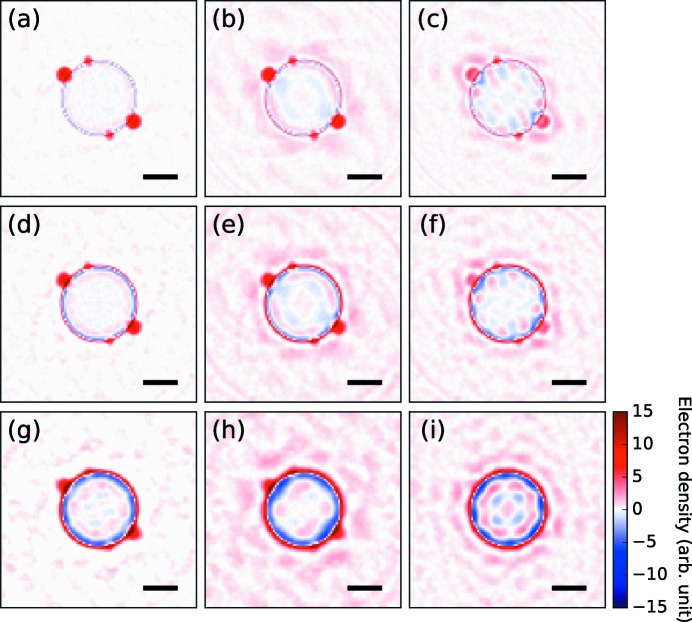
Electron density reconstructed from the simulation data. The initial electron density [Fig. 2 ▸(*b*)] was subtracted from the reconstructed results (Fig. 4 ▸). The scale bars are 100 nm.

Fig. 4 ▸ shows the electron densities reconstructed from the simulated diffraction patterns. To emphasize the changes after the iteration, we subtracted the electron density of the initial structure [*i.e.* the uniform sphere in Fig. 2 ▸(*b*)] from the reconstructed electron density (Fig. 5 ▸). In most of the reconstruction results, we can see the two small spheres that we assumed in the original structure [Figs. 2 ▸(*a*) and 2 ▸(*c*)]. However, the reconstructed structure has two additional spheres at its centrosymmetric positions. Furthermore, the 3D density of each small sphere was equal to or less than half of the 3D densities of each corresponding sphere of the actual object.

The appearance of the twinned objects at centrosymmetric positions is a well known artifact observed in structure reconstruction. A similar problem in the HIO algorithm has also been reported (Fienup, 1982 ▸) and it is called image twinning. This problem stems from the fact that an electron density *f*(*x*, *y*) and its inverted electron density *f*(−*x*, − *y*) give identical diffraction patterns. In the present case, as the initial structure (a uniform dense sphere) has centrosymmetry, the gradient of the likelihood function is also centrosymmetric. As a result, both spheres are reconstructed simultaneously. This problem can be interpreted as a stagnation of the optimization at a saddle-point solution. The present method is unable to avoid the issue, its drawback being that it potentially leads to model-biased results. Therefore, we must note that the present method can be used as a structure refinement method. Future work must focus on developing a better optimization algorithm that can escape from stagnation.

Aside from the problem of image twinning, the refinement method is tolerant to the large missing region of the detector as well as the noise due to the limited number of photons. Using the reconstruction results, we can discuss the qualitative effects on the reconstructed structure caused by the missing region and the limited number of photons. When the missing region of the detector becomes larger, the electron density of the reconstructed structure becomes less accurate. Moreover, the number of diffracted photons limits the spatial resolution of the reconstruction.

Iterative PR problems require constraints, without which they are ill posed and unable to recover unique phases. The most common and powerful constraint is the support region of the object. Outside the support region the electron density is constrained to be zero. However, in our method the choice of the support region did not cause significant changes in the reconstructed structure. This was probably because the updated structure was successfully trapped around the initial structure. Therefore, during the refinement, we did not impose an explicit support region in real space.

To compare the present method with the existing PR algorithms, we applied the ER algorithm starting from the simulated density map shown in Fig. 2 ▸(*b*) (see the Supporting information for details). This combined algorithm successfully reconstructed density maps close to those from the present refinement method. By comparing the density maps reconstructed by the present method and those by the ER algorithm with the initial estimate, we found that the present method is advantageous for the achievement of a better spatial resolution compared with the ER algorithm. If one supposes that the present method is used as a refinement method, these differences in resolution between the two algorithms would not be negligible. We also applied the HIO + ER algorithm starting from a random phase map but could not retrieve any physically meaningful maps.

## 4. Results and discussion   

**Figure 6:**
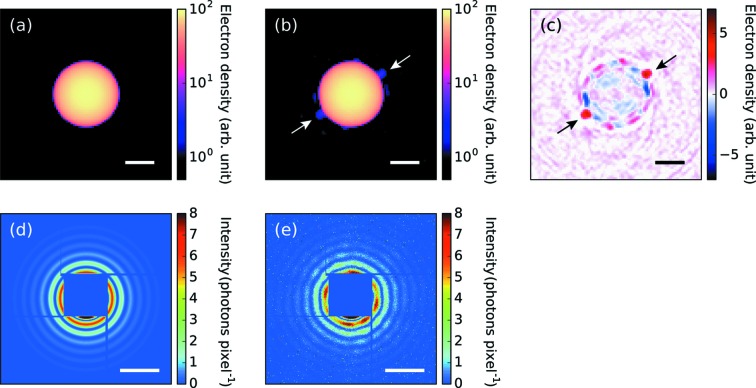
Result of the structure reconstruction from the measured diffraction pattern [Fig. 1 ▸(*c*)] using the implemented algorithm. (*a*) A projection of a uniform dense sphere used as the initial estimate of the density map. (*b*) The reconstructed density map by the implemented algorithm. (*c*) The difference between (*a*) and (*b*) defined as (*b*) − (*a*). (*d*) The simulated pattern from (*a*). (*e*) The simulated pattern from (*b*). The scale bars in (*a*)–(*c*) are 100 nm and those in (*d*)–(*e*) are 0.1 nm^−1^.

We now turn to the experimental data. Fig. 6 ▸ shows the reconstruction result obtained using the experimental data presented in Fig. 1 ▸(*c*). We started the structure refinement with a uniform dense sphere [Fig. 6 ▸(*a*)]. The diffraction pattern calculated from the reconstructed electron density [Fig. 6 ▸(*e*)] reproduced the observed diffraction pattern well, including the characteristic intensity modulation in each ring. The reconstructed electron density indicates a couple of small spheres attached on the surface of the large sphere (indicated by arrows). As discussed above, the twinned structure is probably an artifact of the reconstruction and the object only has one small sphere protruding from the large sphere. This supposition is supported by the fact that the 3D density of the two small spheres is less than half of the 3D density of the large sphere.

**Figure 7:**
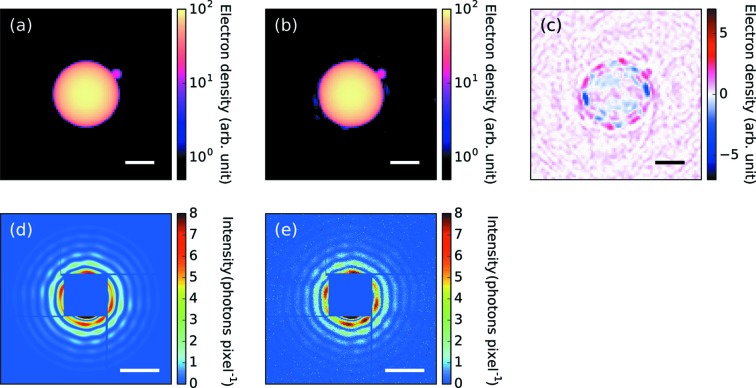
Result of the structure reconstruction from the measured diffraction pattern [Fig. 1 ▸(*c*)] using the implemented algorithm. (*a*) A projection of a sphere with a small sphere used as the initial estimate of the density map. The initial estimate of the density map was constructed from the optimization result shown in Fig. 2 ▸. (*b*) The reconstructed density map by the implemented algorithm. (*c*) The difference between (*a*) and (*b*) defined as (*b*) − (*a*). (*d*) The simulated pattern from (*a*). (*e*) The simulated pattern from (*b*). The scale bars in (*a*)–(*c*) are 100 nm and those in (*d*)–(*e*) are 0.1 nm^−1^.

Here, we tried to avoid the problem of image twinning by starting the iteration with a large sphere coupled with a single small sphere [one-small-sphere structure, Fig. 7 ▸(*a*)]. The radius and position of the small sphere were determined from the previous reconstruction result [Fig. 6 ▸(*c*)] and its 3D density was assumed to be the same as that of the large cluster. The optimization result is shown in Fig. 7 ▸. The optimized value of the Poisson likelihood was −0.131 photons pixel^−1^ and slightly better than that of the previous optimization (−0.138 photons pixel^−1^), which also emphasizes the superiority of the one-small-sphere structure.

The reconstructed electron density is composed of one large sphere attached to a small sphere. The estimated radii of the large and small spheres are 130 nm and 20 nm, respectively. Although a structure similar to this one has been reported in earlier studies involving X-ray scattering experiments using soft XFELs (Rupp *et al.*, 2012 ▸, 2014 ▸), we succeeded in determining the size and shape of the nonspherical cluster using hard XFEL pulses.

## 5. Conclusion   

We developed a refinement method for structure reconstruction that combines the optimization algorithm based on the gradient search method with an initial estimate of the electron-density map. The method was applied to an experimental diffraction pattern from a large Xe cluster. Using a good initial estimate of the electron density, we successfully retrieved the 2D electron-density map even from a low-intensity diffraction pattern without its central region. Our method will enable exploration of the structures of biomolecules and small nanoparticles that do not have high scattering power.

The structure refinement method suggests that prior information on the structure considerably expands the possibilities for structure reconstruction. In fact, we can use many types of prior information to construct the initial structure. In this research, the initial structure was estimated from the diffraction pattern itself. This approach will work when the object closely resembles simple structures such as spheroids or polyhedrons. If physical/chemical constraints can be imposed on the structure, the method could be a powerful tool for structure determination. The present method would be most effective when one is interested in studying small changes occurring in known structures. In this view, the present refinement method is potentially applicable in the study of possible small dynamical structural changes in biomolecules induced by femtosecond optical lasers. 

## Supplementary Material

The results of applying the error reduction algorithm with an initial guess to the simulated diffraction patterns. DOI: 10.1107/S2052252519014222/cw5023sup1.pdf
