# Supplementary material for: Refinement for single-nanoparticle structure determination from low-quality single-shot coherent diffraction data
Source: IUCrJ. 2020 Jan 1;7(Pt 1):10–7. doi: 10.1107/S2052252519014222 (PMC6949595; doi:10.1107/S2052252519014222)
Supplement: Supplementary file 1 [file m-07-00010-sup1.pdf]

IUCrJ

Volume 75 (2019)

Supporting information for article:

Refinement of single nanoparticles structure determination from low-quality single-shot coherent diffraction data

Toshiyuki Nishiyama, Akinobu Niozu, Christoph Bostedt, Ken R. Ferguson, Yuhiro Sato, Christopher Hutchison, Kiyonobu Nagaya, Hironobu Fukuzawa, Koji Motomura, Shin-ichi Wada, Tsukasa Sakai, Kenji Matsunami, Kazuhiro Matsuda, Tetsuya Tachibana, Yuta Ito, Weiqing Xu, Subhendu Mondal, Takayuki Umemoto, Christophe Nicolas, Catalin Miron, Takashi Kameshima, Yasumasa Joti, Kensuke Tono, Takaki Hatsui, Makina Yabashi and Kiyoshi Ueda

Refinement of single nanoparticles structure determination from low-quality single-shot coherent diffraction data
Supporting material

Toshiyuki Nishiyama*,[a,b] Akinobu Niozu*,[a,b] Christoph Bostedt,[c,d,e,f]
Ken R. Ferguson,[c] Yuhiro Sato,[a] Christopher Hutchison,[a]
Kiyonobu Nagaya,[a,b]* Hironobu Fukuzawa,[b,g] Koji Motomura,[e]
Shin-ichi Wada,[b,h] Tsukasa Sakai,[a] Kenji Matsunami,[a]
Kazuhiro Matsuda,[a] Tetsuya Tachibana,[e] Yuta Ito,[e] Weiqing Xu,[e]
Subhendu Mondal,[e] Takayuki Umemoto,[h] Christophe Nicolas,[i]
Catalin Miron,[i,j,k] Takashi Kameshima,[l] Yasumasa Joti,[l] Kensuke Tono,[l]
Takaki Hatsui,[b] Makina Yabashi[b] and Kiyoshi Ueda[b,e]

[a]*Division of Physics and Astronomy, Kyoto University, Kyoto 606-8501, Japan,* [b]*RIKEN SPring-8 Center, Sayo, Hyogo 679-5148, Japan,* [c]*Linac Coherent Light Source, SLAC National Accelerator Laboratory, Menlo Park, CA 94025, USA,* [d]*Chemical Sciences and Engineering Division, Argonne National Laboratory, 9700 S. Cass Avenue, Lemont IL 60439, United States of America,* [e]*Paul-Scherrer Institute, CH-5232 Villigen PSI, Switzerland,* [f]*LUXS Laboratory for Ultrafast X-ray Sciences, Institute of Chemical Sciences and Engineering, École Polytechnique Fédérale de Lausanne (EPFL), CH-1015 Lausanne, Switzerland,* [g]*Institute of Multidisciplinary Research for Advanced Materials, Tohoku University, Sendai 980-8577, Japan,* [h]*Department of Physical Science, Hiroshima University, Higashi-Hiroshima 739-8526, Japan,* [i]*Synchrotron SOLEIL, L'Orme des Merisiers, Saint-Aubin, Cedex*

*Contributed equally

91190, France, [j]*Extreme Light Infrastructure - Nuclear Physics (ELI-NP), "Horia Hulubei" National Institute for Physics and Nuclear Engineering, 30 Reactorului Street, Măgurele RO-077125, Jud.Ilfov, Romania,* [k]*LIDYL, CEA, CNRS, Université Paris-Saclay, CEA Saclay, 91191 Gif-sur-Yvette, France, and* [l]*Japan Synchrotron Radiation Research Institute (JASRI), Sayo, Hyogo 679-5198, Japan.*

E-mail: nagaya@scphys.kyoto-u.ac.jp

1. Application of the error reduction algorithm to the simulated diffraction patterns

In our work, we proposed a refinement method for structure reconstruction from a diffraction pattern and demonstrated it with simulated diffraction patterns. We found that, with a good initial guess of structure, the present method could retrieve a physically meaningful density map from a low-intensity diffraction pattern lacking the center of the image, while we could not reconstruct any physically reasonable density maps with the standard phase retrieval (PR) algorithms. Here we report the typical results of the application of the error reduction (ER) algorithm (Fienup, 1982) with an initial guess of structure to the simulated diffraction patterns shown in Figure 3 of the main text. Then we compare them to the reconstructed density map by using the refinement method described in the main text.

1.1. Scheme of phase retrieval

We defined the density map at the n-th step of iteration by $\rho_n(\vec{r})$, where $(\vec{r})$ was a coordinate in the real space. For updating $\rho_n(\vec{r})$ to $\rho_{n+1}(\vec{r})$, we took the following updating loop:

1. Fourier transform: $\psi_n = \mathcal{F}[\rho_n(\vec{r})](\vec{q})$,

2. constraint in the momentum space: $\tilde{\psi}_n = P_{mom}(\psi_n)$,

3. inverse Fourier transform: $\tilde{\rho}_n = \mathcal{F}^{-1}(\tilde{\psi}_n)$,

4. constraint in the real space: $\rho_{n+1} = P_{real}(\tilde{\rho}_n)$.

Here $\vec{q}$ was a coordinate in the Fourier space. P_{mom} was defined by

$$P_{mom}(\psi_n(\vec{q})) = \begin{cases} \sqrt{|I(\vec{q})|}e^{\arg \psi_n(\vec{q})} & (\vec{q} \in S) \\ \psi_n(\vec{q}) & (\text{otherwise}) \end{cases}, \tag{1}$$

where S was the region where the diffraction intensity $I(\vec{q})$ was known. P_{real} for the ER algorithm was defined by

$$P_{real}(\tilde{\rho}_n(\vec{r})) = \begin{cases} \mathcal{R}e(\tilde{\rho}_n(\vec{r})) & (\mathcal{R}e(\tilde{\rho}_n(\vec{r})) \geq 0) \\ 0 & (\text{otherwise}) \end{cases}, \tag{2}$$

where $\mathcal{R}e$ was the operator which took the real part of a value. The trial of PR using the ER algorithm starts from a projection of a uniformly dense sphere shown in Figure 2b of the main text. The number of iterations of the above update loop was set to 20.

1.2. Results of Phase retrieval

Figure 1 shows the electron density maps reconstructed from the simulated diffraction patterns shown in Figure 3 of the main text by using the combination of the ER algorithm with the initial guess (Figure 2b of the main text). This combined algorithm reconstructed density maps close to those from the present refinement method, which is shown in Figure 4 of the main text. Figure 2 shows the differences of the electron density maps shown in Figure 1 from the initial guess. From Figure 2, and Figure 5 of the main text, it was found that the present refinement method could retrieve the density maps better than the combination of the ER algorithm and the initial guess as the diffraction intensity got lower and the missing region got larger. By comparing Figure 1 with Figure 4 of the main text, we found that the present method is advantageous in the spatial resolution compared with the ER algorithm.

References

Fienup, J. R. (1982). *Appl. Optics*, **21**, 2758–2769.
URL: *https://doi.org/10.1364/AO.21.002758*

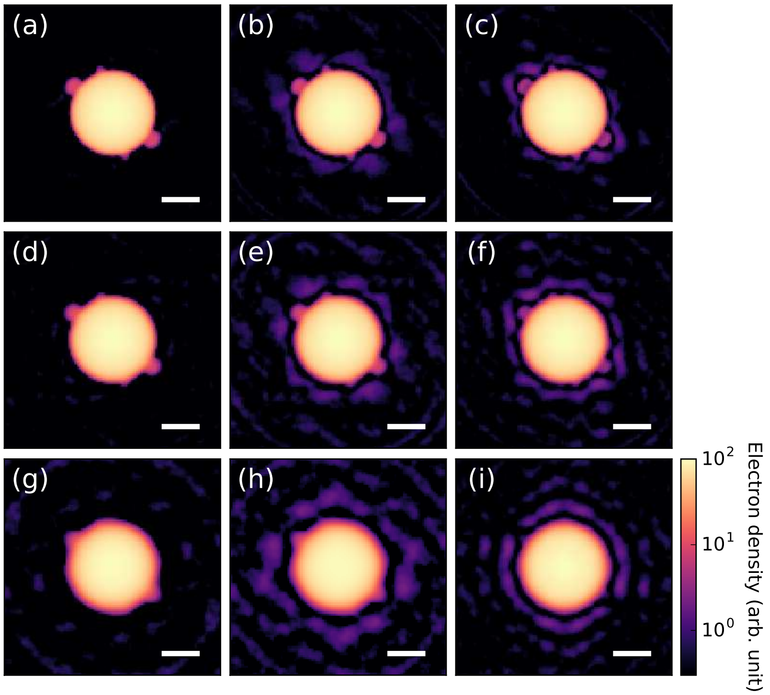

Fig. 1. Electron density reconstructed from the simulation data by using the error reduction algorithm. The density map shown in Figure 2b of the main text was used as the initial guess. Each figure represents the reconstructed structure obtained from the corresponding diffraction pattern in Figure 3 of the main text . The scale bars are 100 nm.

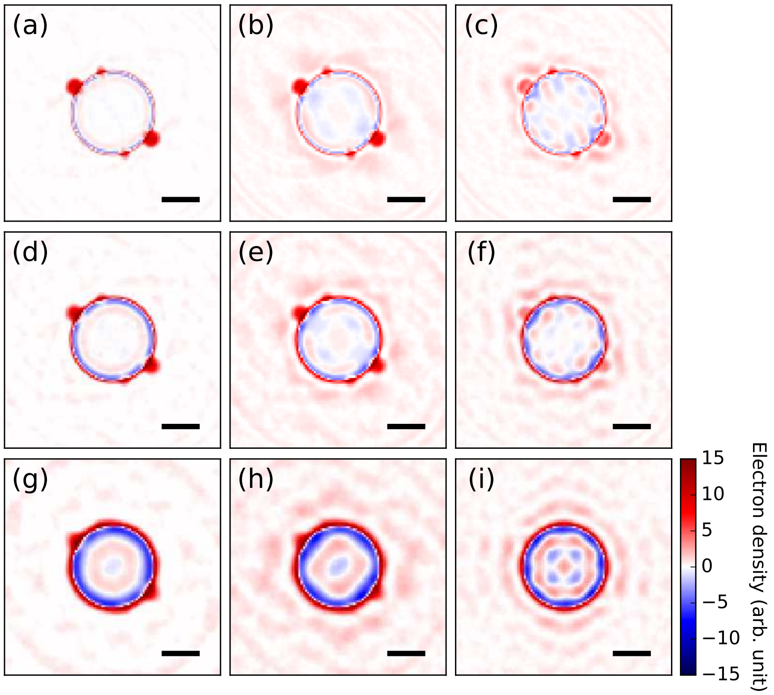

Fig. 2. The difference of the reconstructed electron density by the ER algorithm from the initial guess. The initial electron density (Figure 2b of the main text) was subtracted from the reconstructed results (Figure 1). The scale bars are 100 nm.
